# Expression of NF-κB-p65 and α-SMA in the Study of Capsules formed by Surface Textured Implants Versus Foam Covered Silicone Implants in a Rat Model

**DOI:** 10.29252/wjps.10.3.34

**Published:** 2021-09

**Authors:** Rafael dib Possiedi, Lee Seng Khoo, Francesco Mazzarone, Cleber Rafael Viera da Costa, Patricia Stremel

**Affiliations:** 1 Department of Plastic & Reconstructive Surgery, Hospital Santa Casa de Misericórdia do Rio de Janeiro, 38th Infirmary Professor Ivo Pitanguy’s Service, Rio de Janeiro, Brazil.; 2 Department of Burns & Plastic Surgery, Al Wakra Hospital, Hamad Medical Corporation, Doha, Qatar.; 3 Department of Plastic & Reconstructive Surgery, Skin Check Malaysia, Selangor, Malaysia.; 4 Division of Biological Sciences University of Parana, Parana, Brazil.; 5 Saint Claire Pathology & Cytopathology Labs of Parana, Parana, Brazil

**Keywords:** Mammaplasty, Breast implantation, Implant capsular contracture

## Abstract

**BACKGROUND:**

We aimed to compare inflammatory and intercellular transcription responses induced by surface textured (ST) implants versus foam covered (FC) silicone implants placed on the dorsal aspect of rats.

**METHODS:**

We utilized 80 female rats of the Wistar lineage. The rats were divided into four subgroups of 20 with one type of implant placed in the dorsum per rat. Analysis was carried out on peri-implant capsules at 90 d and at 180 d post-surgery with microscopic evaluation of inflammatory and immuno-histochemical response of NF-κB-p65 and α-SMA in fibroblasts. This study was carried out at the Evangelical Faculty of Parana and at the Ivo Pitanguy Institute, Brazil in 2015.

**RESULTS:**

The FC exhibited higher levels of acute and chronic inflammation on evaluation in both time frames. The capsule surrounding the ST implants was significantly thicker with well-organized collagen fibres. NFκB-p65 expression in the capsule surrounding the FC implant was more pronounced. There was higher and more significant α-SMA expression in the capsules of the surface textured (ST) silicone implants compared to the foam-covered (FC) silicone implants.

**CONCLUSION:**

Activation of NFκB-p65 plays a key role in the evolution of capsule formation and maintenance of inflammation by regulating the healing process. Similarly, higher and more prolonged levels of inflammation (increased NF-κB-p65 results in increased inflammation) and lower α-SMA (higher α-SMA is protective against capsular contracture) did not directly translate to a thicker capsule and ultimately, capsular contracture in foam covered silicone implants.

## INTRODUCTION

Surgically implanted material are foreign bodies that become encapsulated over time. In breast augmentation, a markedly contracted capsule may form secondary to complex interactions mediated by myofibroblasts, inflammatory cells, and extracellular matrix constituents^1^. Capsular contracture remains the most common complication following breast augmentation with implants not infrequently requires implant exchange^2^. The use of surface textured implants has been traditionally thought to decrease the rate and incidence of capsular contracture^3^^-^^6^. 

Gasperoni et al. reported a capsular contracture rate of 3.3% with polyurethane breast implants^7^. Polyurethane covered implants may circumvent the development of capsular contracture. Polyuerthane implants have interconnected, irregular coating forming a spongy meshwork. In contrast of forming a single longitudinal collagen capsule, collagen is deposited conforming to the irregular spongy shape of the coating. Tension from the fibrosis is hence dispersed evenly instead of extending in one linear direction, reducing the incidence of capsular contracture^8^. Nonetheless, both textured and polyurethane implants have now been controversially implicated in the development of Breast Implant Associated – Anaplastic Large Cell Lymphoma (BIA- ALCL)^6^. Wagenführ Jr advocated silicone implants with a silicone foam covering that confer properties similar to polyurethane implants minus the drawbacks such as lamina degradation and possible toxicity of catabolites such as 2-4 TDA (toluenediamine)^9^

NF-κB-p65 (nuclear factor kappa-light-chain-enhancer of activated B cells subtype p-65) is a protein complex that controls transcription of DNA, cytokine production and cell survival^10^. NF-κB-65 controls many genes involved in inflammation. A disproportionate increase in activated NF-κB-p65 is key to the pathogenesis of many chronic diseases^10^ and is present in capsule tissue of implants.

α-SMA is used as a biomarker of myofibroblasts, it also potentiates the contractile activity of myofibroblasts^11^. Contracted capsules exhibit more collagen fibre alignment and α-SMA-positive immunoreactivity than uncontracted capsules (Baker I and II)^12^. Capsules from textured implants also show less α-SMA-positive immunoreactivity compared to capsules from smooth implants^12^. α-SMA is the actin isoform that predominates within vascular smooth-muscle cells and plays a key role in fibrosis^11^ and hence capsular contracture.

The aim of this study was to elicit the process of capsular contracture and compare inflammatory and intercellular transcription responses (namely the microscopic evaluation of inflammatory and immuno-histochemical response of NF-κB-p65 and α-SMA in fibroblasts) induced by surface textured (ST) implants versus foam covered (FC) silicone implants utilizing a rat model.

## METHODS

This study was performed at the Research Laboratory of the Institute of Medical Research (IMR) at the Evangelical Faculty of Parana in cooperation with the Ivo Pitanguy Institute, after being approved by the Ethics Committee on the Use of Animals at the Evangelical Faculty of Parana and the Ivo Pitanguy Institute registered under number 11678/2014, from Jan 2015 to Jul 2015.

Eighty female Wistar rats (Rattus norvegicus albinus , Roentia mammalia),100 to 120 d of life with a median weight of 240 g supplied by the Vivarium of the Health Sciences Sector of the Federal University of Paraná were utilized for the study. They were distributed in acrylic boxes measuring 500 cm3 containing four rats in each with free access to water and specific diet for the species, with a 12-hour dark/light cycle and central air conditioning (18 °C, 60% humidity).

The rats were divided into four subgroups of 20 with one type of implant placed in the dorsum per rat (Table 1). Analysis was carried out on peri-implant capsules at 90 d and at 180 d post-surgery with macroscopic measurement of capsule thickness and microscopic evaluation of inflammatory and immuno-histochemical response of NF-κB-p65 and α-SMA in fibroblasts.

***Experimental Procedure***

The rats were anesthetized with Ketamine (Ketalar®, Aché Laboratórios Farmacêuticos AS, São Paulo, Brasil) 100mg/Kg, combined with Chlorpromazine 10 Virbaxyl 2%®, Virbac do Brasil, São Paulo, Brasil) 10mg/Kg via intraperitoneal injection. Prior to surgery, the dorsums of the anesthetized rats were shaved and antisepsis achieved with Chlorhexidine solution.

**Fig. 1:**
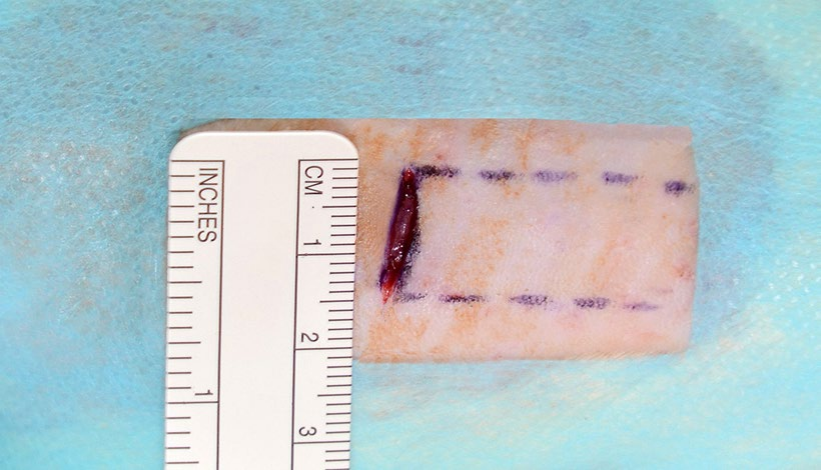
Horizontal skin incision and pre-marked area of dissection

The site of incision was standardized to the intersection between the horizontal line along the postero-inferior costal margin to the sagittal midline. A horizontal incision was made using a number 15 blade with an extension of 1.5 cm at the intersection of these imaginary lines (Figure 1).

**Fig. 2:**
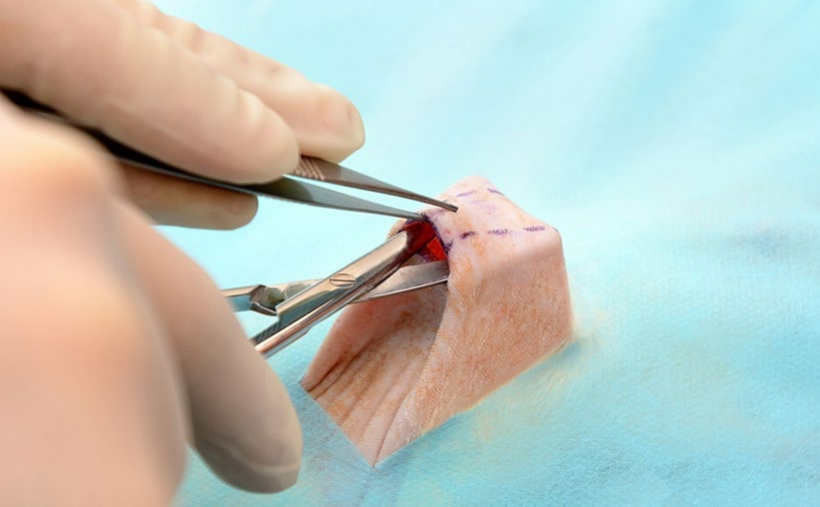
A Subcutaneous pocket extending cranially was dissected with scissors to allow placement of the implants

A subcutaneous pocket extending cranially was dissected with scissors to allow placement of the implants (Figure 2).

**Fig. 3:**
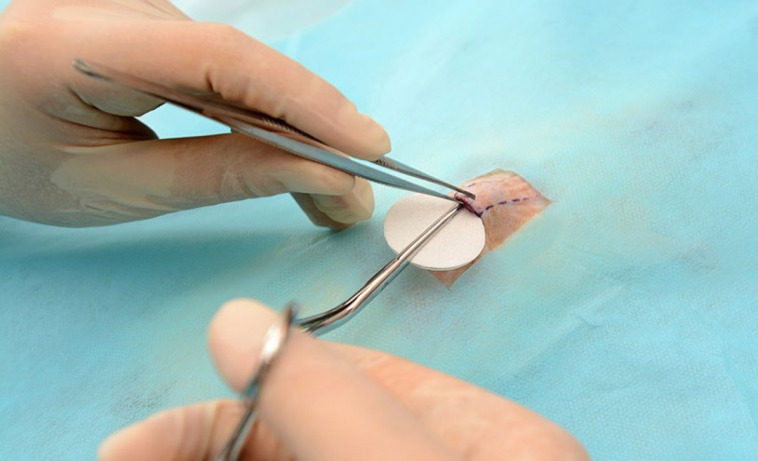
A Magill Forceps was used to place the implants into the predissected space

**Fig. 4:**
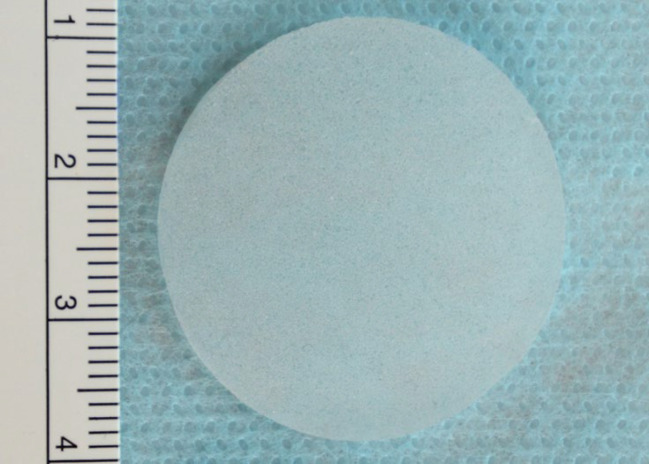
Surface Textured (ST) discoid shaped implant

**Fig. 5:**
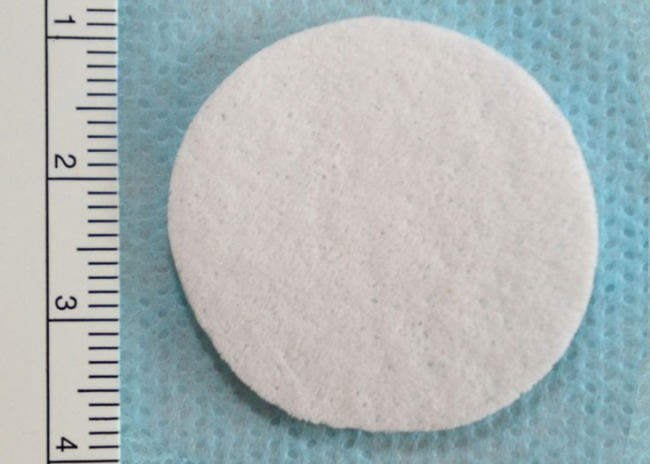
Foam Covered (FC) discoid shaped implant

A Magill Forceps was used to place the implants into the predissected space (Figure 3). Each subgroup received only one type of implant either Surface Textured (ST) (Figure 4) or Foam Covered (FC) (Figure 5). Each implant was standardized to 3 cm in diameter.

After placement of implants, the wound was closed with interrupted simple 4/0 Nylon sutures. The wounds were kept open with no post-operative dressings or suture removal. The rats were sacrificed according to their subgroups at the predetermined time frame with Sodium Thiopental (90 mg/kg) administered intraperitoneally. 

**Fig. 6:**
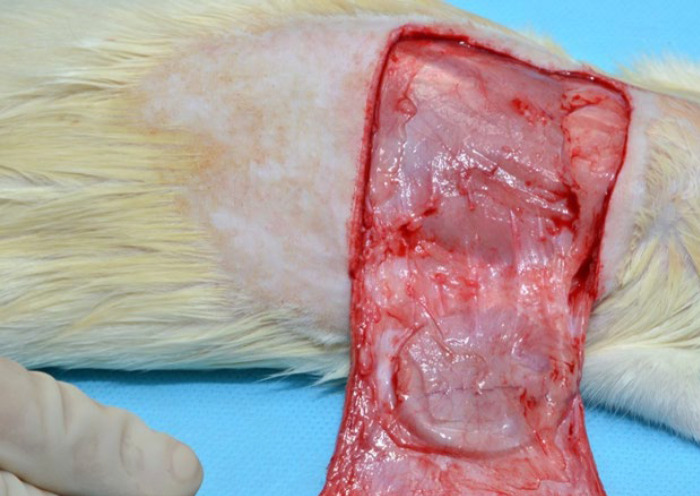
The silicone implants were removed en-bloc with their capsules

The silicone implants were removed en-bloc with their capsules (Figure 6) and submitted for immunohistological study. All slides were evaluated by the same pathologist, blinded to the group or time frame to which the specimen belongs.

***Histological evaluation***

The specimens were fixed with 10% PBS (PBS® 10x, Irvine Scientific) and buffered formalin (Formalin®, Richard-Allan). They were dehydrated and embedded in the paraffin before being sectioned for slide examination of protein expression via immunohistochemistry staining. 

The sections were incubated for 12 h at 4 ºC for detection of primary antibodies. The primary antibodies used were anti-NfκB p65 (Santa Cruz Biotechnology®, CA, SC-372, rabbit IgG dilution 1:25) and anti-smooth muscle actin (Dakocytomation® M0851, mouse IgG, dilution 1: 400). At end of the incubation period, the slides were again immersed in a polymer detection kit that identifies and binds to Easylink Duo rabbit and mouse antibodies (Easypath EP-12-24094®).

Following of which the slides were once again dehydrated by ethanol and xylene and mounted with Entellan® (Merck®). The results were examined and photographed under optical microscopy.

***Hematoxylin-eosin staining***

With hematoxylin-eosin staining, the following histological variables were evaluated: Polymorphonuclear leukocytes (PMNs) signalling acute inflammation and macrophages/lymphocytes signalling chronic inflammation. The presence of these cells and other subcellular types was graded as follows: absent, mild, moderate and severe.

***Capsule thickness measurement***

The thickness of the capsule was measured at three points on each slide. The average of these measurements corresponded to the thickness of that slide.

***Statistical Analysis***

Statistical analysis was performed with Analysis of Variance (ANOVA) aiming to evaluate the influence of the type of implant (ST vs PF) and the time frame (90 and 180 d). Shapiro-Wilks test was used to evaluate data normality. Statistical significance was set at *P*<0.05. The software utilized was SAS 6.11 software (SAS Institute, Inc., Cary, North Carolina).

## RESULTS

***Hematoxylin-eosin staining***

**Fig. 7:**
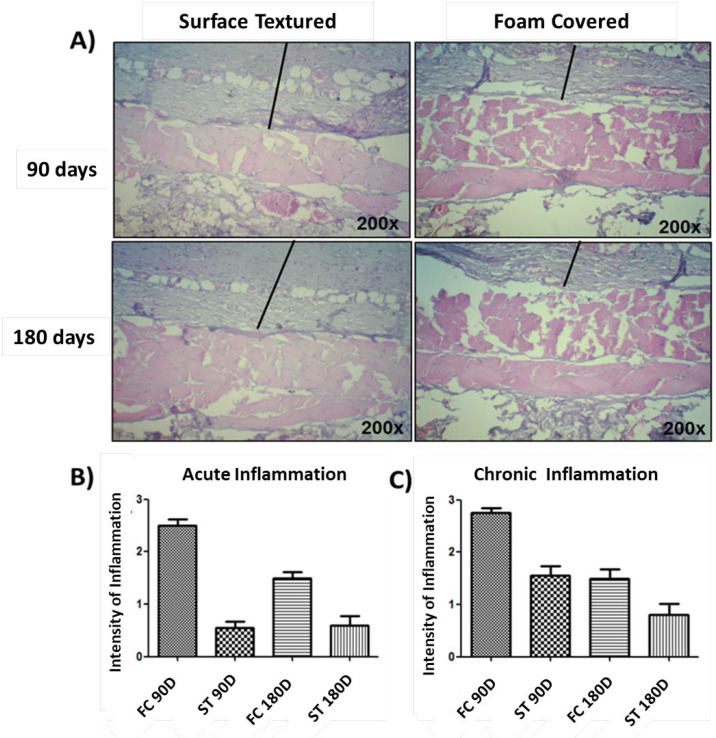
Images of pericapsular tissue inflammation 200x magnification. A) Histological changes in each sample were analyzed with hematoxylin-eosin staining of tissue sections, which were graded from 0 to 4. Graphical representation of acute inflammation (B) and chronic inflammation (C) in the capsules surrounding the implants. The results were expressed as median ± SD. p <0.0001 (ANOVA and Shapiro-Wilks)

Foam covered (FC) silicone implants exhibited greater degrees of both acute and chronic inflammatory reactions compared to surface textured implants during both intervals of assessment (*P*=<0.0001) (Figure 7).

***Immunohistochemistry***

***Nuclear factor kappa B (NFκB-p65)***

**Fig. 8:**
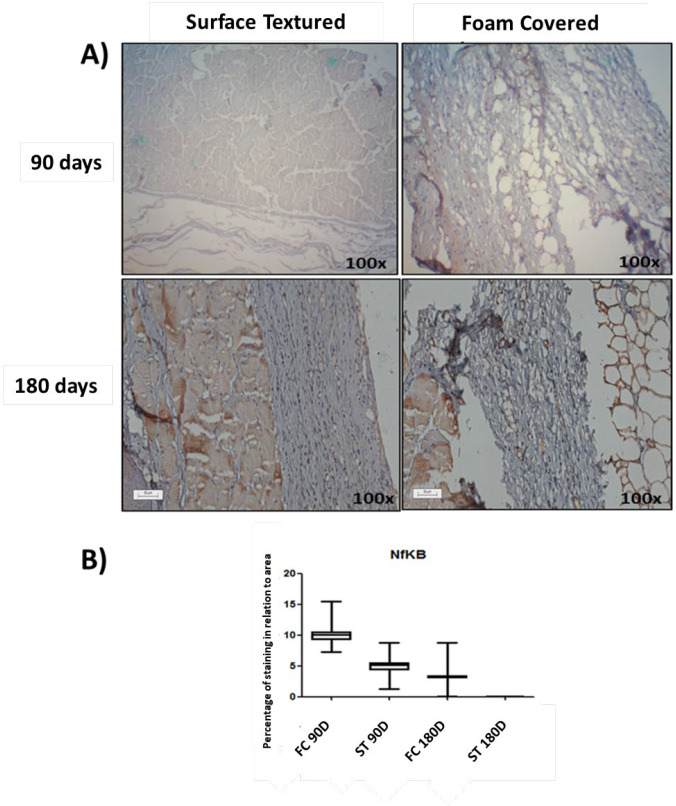
(A) Representative images of NFκB-p65 expression in peri-implant tissue. Magnification 100x. (B) Graphical representation of the percentage in relation to the immunohistochemical expression area obtained through the analysis of the images of the peri-implant capsule and the results were expressed as median ± SD. *P*<0.001 (ANOVA and Shapiro-Wilks)

The intensity of NFκB-p65 expression in the capsule covered with foam implant was more pronounced than on the surface textured implants 90 and 180 d after surgery (*P*=<0. A) (Figure 8).

***Alpha-smooth muscle actin (alpha-SMA)***

**Fig. 9:**
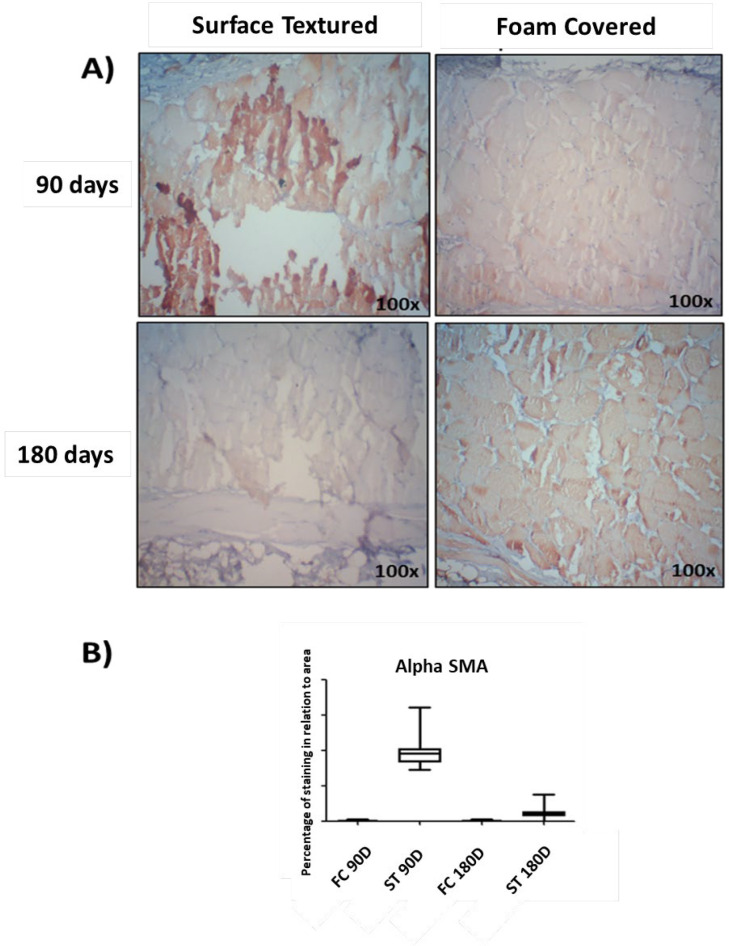
(A) Representative images of the expression of α-SMA (a marker for myofibroblasts) in peri-implant tissue. Magnification 100x. (B) Graphical representation of the percentage of immunohistochemical expression obtained through the analysis of the images of the peri-implant capsule and the results were expressed as median ± SD. *P*<0.001 (ANOVA and Shapiro-Wilks)

Immunohistochemistry revealed greater expression of α-SMA in the capsules of surface textured implants, when compared to foam covered implants, both at 90 d (*P*=<0.0001) and 180 d (*P*=<0.0001) after surgery (Figure 9).

Immunohistochemical analysis for α-SMA in the capsular tissue around the textured and silicone foam surfaces.

***Capsule Thickness***

**Fig. 10:**
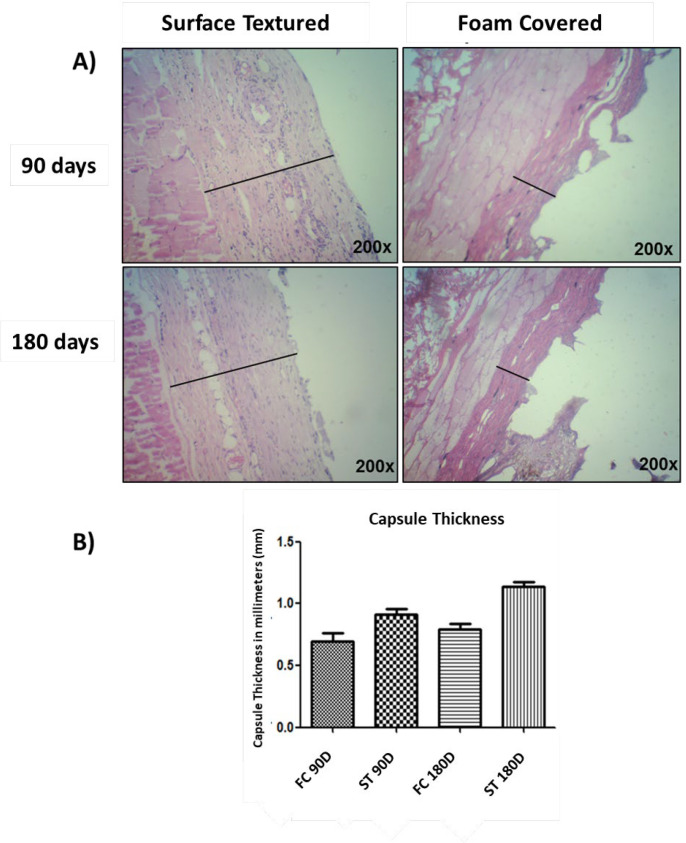
The capsules surrounding the textured implants were significantly thicker when compared to foam covered implants at 90 d (*P*=0.0001) and 180 d (*P*<0.0001)

The capsules surrounding the textured implants were significantly thicker when compared to foam covered implants at 90 d (*P*=0.0001) and 180 d (*P*<0.0001) (Figure 10). In addition, the collagen present in the peri-implant layer in textured implants appear more continuous and parallelly aligned than in foam covered implants.

## DISCUSSION

***Inflammatory Reactions***

Placement of silicone implants in tissues typically induces an inflammatory response characterized by an infiltrate consisting of macrophages, foreign body giant cells, and a variable number of plasma cells and lymphocytes which initiates fibrosis^13^. Fibrosis is a process triggered by complex reactions in vivo normally induced by foreign body material. It serves as a defence mechanism in response to infection, foreign body material and autoimmune factors amongst others^14^.

Stages of fibrosis are as follows: 1. blood-biomaterial interaction, 2. provisional matrix formation, 3. acute inflammation, 4. chronic inflammation, 5. foreign body giant cell formation, and 6. fibrous capsule formation^15^. In theory, the longer the duration of inflammation be it acute or chronic, the more pronounced the foreign body reaction resulting in a thicker capsule and higher grade of capsular contracture. Acute inflammation is characterized by the presence of polymorphonuclear cells, predominantly neutrophils, while chronic inflammation presents lymphocytes, monocytes, vascular proliferation and fibrosis^16^.

Balderrama reported mild or absent acute inflammatory reaction in all groups (7, 14, 30, 60 d) in the analysis of the reaction to textured and silicone foam coatings^17^.

In contrast, our study demonstrated that the foam-covered group had moderate (55%) and intense (45%) inflammatory reactions within 90 days. The 180-day analysis of the foam-covered group, the reaction was mild in 50% and moderate in the other 50% of the sample. However, these reactions were absent in 45% and mild in 55% on day 90 evaluation of the textured surface group. In the 180-day samples from the textured group, they showed absent (55%), mild (30%) and moderate (15%) reactions.

Regarding the chronic inflammatory reaction, there was a predominance of mild (50%), followed by moderate (30%) and intense (15%) in the surface textured groups at 90 d, contrary to the findings of Lesesne^18^ who did not find intense inflammation in the tissue reaction to the surface textured implant. However, with 180 d in the surface textured group, the reaction became minimal in 50%, followed by mild (25%), moderate (20%) and severe in only 5%.

These data partially concur with the findings that reported minimal reactions for textured implants^19^. Contrary to the findings of Wagenführ Jr, who observed absence or minimal presence in all animal groups in the four evaluation periods (28 d, 2 months, 3 months and 6 months)^20^, severe chronic inflammation was evident in our study for surface textured group in 90 d and evolving to moderate (50%), mild (35%), absent (10%) and remaining severe in 5% of the 180-day sample.

***Immunohistochemistry***

***Nuclear factor kappa B (NFκB-p65)***

NFκB is a transcription factor that play an essential role in inflammation, lymphocyte activation, cell survival and in the formation of secondary lymphoid organs. It is also an important component of lymphocyte development and the pathogenesis of many cancers^21^. Different combinations of NFκB subunits have different roles in the immune response. The transcription of pro-inflammatory genes in the classic NFκB signaling pathway is regulated by the p65 / RelA-p50 heterodimer^9^^,^^10^^,^^22^.

In our study, the expression of the NFκB p65 / RelA subunit gene was significantly higher in the foam covered (FC) silicone groups at both time frames (90 and 180 d) of evaluation. These results provided evidence of the presence of increased local inflammation for a longer duration when compared to surface textured (ST) implants.

To date, there are no studies published in the literature investigating the expression and activation of the classic NFκB pathway, as well as its role in the formation of the capsule or in the contracture mechanism around silicone breast implants. However, its involvement has been widely investigated in fibrotic diseases^23^^,^^24^, mainly in the regulation of fibronectin transcription that induces cell differentiation, migration, coagulation and formation of extracellular matrix, which in the latter analysis serves to promote wound contraction^25^. According to Barnes and Gorin^26^ and Clarke et al.^27^ fibronectin deposition results in fibrosis in several organs.

NFκB is related to the production of metalloproteinases, which degrade matrix macromolecules, including interstitial collagen, fibronectin, laminin and proteoglycan, among others. Collectively, metalloproteinases are able to degrade all proteins that make up the extracellular matrix and basement membranes.

An increase was demonstrated in serum concentrations of metalloproteinase MMP-2 and inhibitors of metalloproteinases TIMP-1 and TIMP-2 in patients with capsular contracture, as well as a decrease in MMP / TIMP ratio, correlating with the severity of Baker’s grade of contracture. There were marked increase in the expression of TIMP in smooth implants, compared to textured implants^28^. The high concentrations of TIMP may be involved in the pathogenesis of capsular contracture, as well as explaining the higher rates of capsular contracture observed in smooth implants.

***α-SMA***

The increased presence of positive α-SMA detection in capsules is an indicative sign of myofibroblast activation resulting in increased contracture in both scar tissue and capsules^29^. Smooth muscle α-actin (α-SMA) induce production of higher amounts of extracellular matrix proteins, such as type I collagen and fibronectin which possesses contractile properties. The prime activators are IL-6 and TGF-β1, although they can also be activated by a variety of other cytokines, chemokines, growth factors, components of microbial cell walls, and by members of the oxidative stress cascade^30^. 

The intensity of α-SMA expression in the capsule around polyurethane-coated implants was higher when compared to textured surface implants at 30 d after surgery. However, the increased intensity was not observed 90 d after implantation^31^.

In our study, the intensity of α-SMA expression in the capsule of surface textured (ST) implants was significantly higher when compared to foam covered (FC) implant surfaces at both 90 and 180 d after surgery.

Fibroblasts can be derived from quiescent connective tissue fibroblasts at proliferation sites, but there is also ample evidence that at least some of them originate from myeloid precursors in the blood or bone marrow that migrate to injury sites^30^.

Positive immunoreactivity for α-SMA has been reported higher than in those without contracture (Baker I and II). Positive immunoreactivity for α-SMA was significantly lower in textured implants as opposed to smooth implants^29^.

Darby et al.^32^ and Skalli et al.^33^ demonstrated myofibroblasts at the capsule device interface had increased immunopositive staining for α-SMA supporting the hypothesis that myofibroblasts play an active role in capsular contracture^34^.

***Capsule Thickness***

The capsule surrounding the ST implants was significantly thicker than the FC implants with well-organized collagen fibres at both 90 and 180 d of our study. This is in contrast to the study that reported thicker capsules in the subgroups of foam-covered silicone implants as opposed to surface textured implants at 7 and 60 days^17^.

Baker I capsules were significantly thinner than Baker II, III, IV and the thickness of the capsule is directly proportional to the length of time for all capsules including those with contracture (Baker III and IV)^29^. Rubino et al.^35^ found thinner capsules in textured implants without capsular contracture and Prantl et al.^36^ found an association between greater capsule thickness and clinical signs of contracture.

Limitations of this study are acknowledged as the study was conducted on an animal model with the limited time frame for removal of the implants for purposes of the study which may not directly translate to the human model. 

Both textured and polyurethane implants although conferring a lower risk of capsular contracture, have been controversially implicated in the development of Breast Implant Associated – Anaplastic Large Cell Lymphoma (BIA- ALCL)^6^. Foam covered silicone implants have not been studied as extensively as its polyurethane coated counterpart. Currently, the only manufacturer of foam-covered silicone implant in the world is LifeSil^TM^ based in Curitiba, Paraná, Brazil. To date there has been no report of BIA-ALCL associated with foam covered silicone implants^37^ Theoretically, the foam’s structure mimics that of polyurethane^9^ and confers the benefits of reduced rate of capsular contracture without the possible toxic metabolites of 2-4 TDA (toluenediamine)^17^. Nonetheless, the higher and more prolonged levels of inflammation (increased NF-κB-p65) and lower α-SMA levels associated with FC implants raises unanswered questions regarding its long-term safety. Now with polyurethane implants being vilified rightly or wrongly^38^, more studies have to be done to determine the safety of foam-covered silicone implants.

**Table 1:** Distribution of groups and subgroups with time frame for explantation & analysis

	**Subgroups**	
**Group**	90 Days	180 Days
**Foam Covered (FC)**	n: 20	n: 20
**Surface Textured (ST)**	n: 20	n: 20

## CONCLUSION

The FC exhibited higher levels of acute and chronic inflammation on evaluation in both time frames. The capsule surrounding the ST implants was significantly thicker with well-organized collagen fibres. NFκB-p65 expression in the capsule surrounding the FC implant was more pronounced. There was higher and more significant α-SMA expression in the capsules of the surface textured (ST) silicone implants compared to FC silicone implants. 

Activation of NFκB-p65 plays a key role in the evolution of capsule formation and maintenance of inflammation by regulating the healing process. Similarly, higher and more prolonged levels of inflammation (increased NF-κB-p65 results in increased inflammation) and lower α-SMA (higher α-SMA is protective against capsular contracture) did not directly translate to a thicker capsule and ultimately more pronounced capsular contracture in foam covered silicone implants. In other words, the severity of capsular formation & contracture does not increase with severity and duration of inflammation.

## CONFLICT OF INTEREST

The authors declare that there is no conflict of interests.

## References

[1] Araco A, Caruso R, Araco F, Overton J, Gravante G (2009). Capsular contractures: a systematic review. Plast Reconstr Surg.

[2] Handel N, Cordray T, Gutierrez J (2006). A long-term study of outcomes, complications, and patient satisfaction with breast implants. Plast Reconstr Surg.

[3] Coleman DJ, Foo LTH, Sharpe DT (1991 ). Textured or smooth implants for breast augmentation? A prospective controlled trial. Br J Plast Surg.

[4] Ersek RA (1991 ). Rate and incidence of capsular contracture: a comparison of smooth and textured Silicone double-lumen breast prostheses. Plast Reconstr Surg.

[5] Pollock H (1993). Breast capsular contracture: a retrospective study of textured versus smooth Silicone implants. Plast Reconstr Surg.

[6] Swanson E (2018). A 1-point plan to eliminate Breast Implant-Associated Anaplastic Large-Cell Lymphoma (BIA-ALCL). Ann Plast Surg.

[7] Gasperoni C, Salgarello M, Gargani G (1992 ). Polyurethane-covered mammary implants: a 12-year experience. Ann Plast Surg.

[8] Brand KG (1988). Foam-covered mammary implants. Clin Plast Surg.

[9] Wagenfuhr Jr J (2007). Comparative Histopathological Analysis of Silicone and Polyurethane Foam Implants Capsules in Rats. Rev Bras Cir Plast.

[10] Giridharan S, Srinivasan M (2018). Mechanisms of NF-κB p65 and strategies for therapeutic manipulation. J Inflamm Res.

[11] Hinz B, Phan SH, Thannickal VJ, Prunotto M, Desmouliere A, Varga J (2012). Recent developments in myofibroblast biology: paradigms for connective tissue remodeling. Am J Pathol.

[12] Bui JM, Perry T, Ren CD, Nofrey B, Teitelbaum S, Van Epps DE (2015). Histological characterization of human breast implant capsules. Aesthetic Plast Surg.

[13] Anderson JM, Rodriguez A, Chang DT (2008). Foreign body reaction to biomaterials. Semin Immunol.

[14] Wick G, Backovic A, Rabensteiner E, Plank N, Schwentner C, Sgonc R (2010). The immunology of fibrosis: innate and adaptive responses. Trends Immunol.

[15] Klopfleisch R, Jung F (2017). The pathology of the foreign body reaction against biomaterials. J Biomed Mater Res A.

[16] Cotran RS, Kumar V, Collins T ( 2014). Robbins pathologic basis of disease.

[17] Balderrama CM, Ribas-Filho JM, Malafaia O, Czeczko NG, Dietz UA, Sakamoto DG, Bittencourt LP (2009). Healing reaction to mammary prostheses covered by textured silicone and silicone foam in rats. Acta Cir Bras.

[18] Lesesne CB (1997). Textured surface silicone breast implants: histology in the human. Aesthetic Plast Surg.

[19] Batra M, Bernard S, Picha G (1995). Histologic comparison of breast implant shells with smooth, foam, and pillar microstructuring in a rat model from 1 day to 6 months. Plast Reconstr Surg.

[20] Wagenfuhr Jr ( 2007). Análise histológica comparativa das cápsulas dos implantes de espumas de silicone em ratos. Revista da Sociedade Brasileira de Cirurgia Plástica.

[21] Abbas AK, Lichman AH, Pillay S ( 2014). Cellular and molecular immunology.

[22] Saccani A, Schioppa T, Porta C, Biswas SK, Nebuloni M, Vago L, Bottazzi B, Colombo MP, Mantovani A, Sica A (2006). p50 nuclear factor-kappaB overexpression in tumor-associated macrophages inhibits M1 inflammatory responses and antitumor resistance. Cancer Res.

[23] Messadi DV, Doung HS, Zhang Q, Kelly AP, Tuan TL, Reichenberger E, Le AD (2004). Activation of NFkappaB signal pathways in keloid fibroblasts. Arch Dermatol Res.

[24] Tabary O, Boncoeur E, de Martin R, Pepperkok R, Clément A, Schultz C, Jacquot J (2006). Calcium-dependent regulation of NF-(kappa)B activation in cystic fibrosis airway epithelial cells. Cell Signal.

[25] Ishise H, Larson B, Hirata Y, Fujiwara T, Nishimoto S, Kubo T (2015). Hypertrophic scar contracture is mediated by the TRPC3 mechanical force transducer via NFkB activation. Sci Rep.

[26] Barnes JL1, Gorin Y (2011). Myofibroblast differentiation during fibrosis: role of NAD(P)H oxidases. Kidney Int.

[27] Clarke DL, Carruthers AM, Mustelin T, Murray LA (2013). Matrix regulation of idiopathic pulmonary fibrosis: the role of enzymes. Fibrogenesis Tissue Repair.

[28] Ulrich D, Ulrich F, Pallua N, Eisenmann-Klein M ( 2009). Effect of tissue inhibitors of metalloproteinases and matrix metalloproteinases on capsular formation around smooth and textured silicone gel implants. Aesthetic Plast Surg.

[29] Bui JM, Perry T, Ren CD, Nofrey B, Teitelbaum S, Van Epps DE ( 2015). Histological characterization of human breast implant capsules. Aesthetic Plast Surg.

[30] Wynn TA ( 2008). Cellular and molecular mechanisms of fibrosis. J Pathol v..

[31] Vieira VJ, d’Acampora AJ, Marcos AB, Di Giunta G, de Vasconcellos ZA, Bins-Ely J, d’Eça Neves R, Figueiredo CP (2010). Vascular endothelial growth factor overexpression positively modulates the characteristics of periprosthetic tissue of polyurethane-coated silicone breast implant in rats. Plast Reconstr Surg.

[32] Darby I, Skalli O, Gabbiani G (1990). Alpha-smooth muscle actin is transiently expressed by myofibroblasts during experimental wound healing. Lab Invest.

[33] Skalli O, Ropraz P, Trzeciak A, Benzonana G, Gillessen D, Gabbiani (1986). A monoclonal antibody against alpha-smooth muscle actin: a new probe for smooth muscle differentiation. G J Cell Biol.

[34] Huang BP, Lin CH, Chen HM, Lin JT, Cheng YF, Kao SH (2015). DNA Cell Biol. AMPK activation inhibits expression of proinflammatory mediators through downregulation of PI3K/p38 MAPK and NF-κB signaling in murine macrophages. DNA Cell Biology.

[35] Rubino C, Mazzarello V, Farace F, D’Andrea F, Montella A, Fenu G, Campus GV ( 2001). Ultrastructural anatomy of contracted capsules around textured implants in augmented breasts. Ann Plast Surg.

[36] Prantl L, Pöppl N, Horvat N, Heine N, Eisenmann-Klein M ( 2007). Clinical and morphological conditions in capsular contracture formed around silicone breast implants. Plast Reconstr Surg.

[37] Ivana Leme dC (2019). Evaluation of Clinical Evolution of Breast Augmentation Using Implants with Silicone Foam Envelope and Implants with Textured Silicone Envelope. Adv Plast Reconstr Surg.

[38] Hamdi M (2019). Association Between Breast Implant-Associated Anaplastic Large Cell Lymphoma (BIA-ALCL) Risk and Polyurethane Breast Implants: Clinical Evidence and European Perspective. Aesthet Surg J.

